# Hyperglycemia Promotes Endothelial Cell Senescence through AQR/PLAU Signaling Axis

**DOI:** 10.3390/ijms23052879

**Published:** 2022-03-07

**Authors:** Yiqi Wan, Zhirui Liu, Andong Wu, Abdul Haseeb Khan, Ying Zhu, Shuangjin Ding, Xueer Li, Ya Zhao, Ximo Dai, Jin Zhou, Jiankun Liu, Yuanyuan Li, Xueting Gong, Man Liu, Xiao-Li Tian

**Affiliations:** Aging and Vascular Diseases, Human Aging Research Institute (HARI), School of Life Science, Jiangxi Key Laboratory of Human Aging, Nanchang University, Nanchang 330031, China; yiqi_wan@email.ncu.edu.cn (Y.W.); liuzhirui2020@outlook.com (Z.L.); andongwu@ncu.edu.cn (A.W.); khan.ncu@outlook.com (A.H.K.); pink-shadow@gmu.edu.cn (Y.Z.); 402404718072@email.ncu.edu.cn (S.D.); lixueer@email.ncu.edu.cn (X.L.); yazhao@email.ncu.edu.cn (Y.Z.); daiximo@email.ncu.edu.cn (X.D.); zhouxiaojinzj@163.com (J.Z.); liujiankun@email.ncu.edu.cn (J.L.); easily008@163.com (Y.L.); gongxt1234@163.com (X.G.); liumander@163.com (M.L.)

**Keywords:** diabetes, AQR, endothelial senescence, PLAU, inflammation

## Abstract

Hyperglycemia is reported to accelerate endothelial cell senescence that contributes to diabetic complications. The underlying mechanism, however, remains elusive. We previously demonstrated *AQR* as a susceptibility gene for type 2 diabetes mellitus (T2DM) and showed that it was increased in multiple tissues in models with T2DM or metabolic syndrome. This study aimed to investigate the role of AQR in hyperglycemia-induced senescence and its underlying mechanism. Here, we retrieved several datasets of the aging models and found the expression of AQR was increased by high glucose and by aging across species, including *C. elegans* (whole-body), rat (cardiac tissues), and monkey (blood). we validated the increased AQR expression in senescent human umbilical vein endothelial cells (HUVECs). When overexpressed, AQR promoted the endothelial cell senescence, confirmed by an increased number of cells stained with senescence-associated beta-galactosidase and upregulation of CDKN1A (P21) as well as the prohibited cellular colony formation and G2/M phase arrest. To explore the mechanism by which AQR regulated the cellular senescence, transcriptomic analyses of HUVECs with the overexpression and knockdown of the AQR were performed. We identified 52 co-expressed genes that were enriched, in the terms of plasminogen activation, innate immunity, immunity, and antiviral defense. Among co-expressed genes, *PLAU* was selected to evaluate its contribution to senescence for its highest strength in the enrichment of the biological process. We demonstrated that the knockdown of PLAU rescued senescence-related phenotypes, endothelial cell activation, and inflammation in models induced by AQR or TNF-α. These findings, for the first time, indicate that AQR/PLAU is a critical signaling axis in the modulation of endothelial cell senescence, revealing a novel link between hyperglycemia and vascular dysfunction. The study may have implications in the prevention of premature vascular aging associated with T2DM.

## 1. Introduction

Diabetes mellitus, featured with hyperglycemia, is a common risk factor for many other diseases, resulting in diabetic complications including nephropathy, retinopathy, neuropathy, and cardiovascular diseases [1], and most of these complications are linked to vascular dysfunction [2,3,4]. It has been reported that hyperglycemia promotes inflammation and the production of reactive oxidative species (ROS) through multiple regulatory mechanisms, impairing vascular cells (endothelial cell and smooth muscle cell) that eventually damage vascular function [3,5,6,7,8,9]. In addition, there is growing evidence that hyperglycemia induces premature senescence in endothelial cells [10], smooth muscle cells [11], endothelial progenitor cells [12], and mesenchymal stem cells [13], suggesting that cellular senescence in vessels may play an important role in the process of diabetic complications. The mechanism by which hyperglycemia promotes senescence is yet to be fully elucidated.

*AQR* is a gene encoding intron-binding protein. Although AQR has been proposed to be involved in RNA splicing, its function has not been well-characterized [14]. An earlier study showed that knockdown of *emb4*, a homologous gene of *AQR*, extends the lifespan of *C. elegans* with energy deficiencies [15]. We have reported *AQR* as a novel susceptibility gene for T2DM, as the expression of AQR was increased in several animal models with T2DM or metabolic syndrome, the alleles associated with decreased expression of AQR exhibited a protective effect, and knockdown of AQR facilitated the glucose uptake and restore the insulin sensitivity [16]. More recently *AQR* has been linked as a susceptibility gene for estimated glomerular filtration rate, whose function largely relies on the integrity of vasculatures in the kidney [17]. These observations support that AQR not only is important to diabetes, but also has the potential to be involved in vascular aging.

In this study, we planned to test a hypothesis that AQR contributes to endothelial cell senescence and to search for the possible mechanisms in different models.

## 2. Results

### 2.1. The Expression of AQR Is Increased with Aging

**Figure 1:**
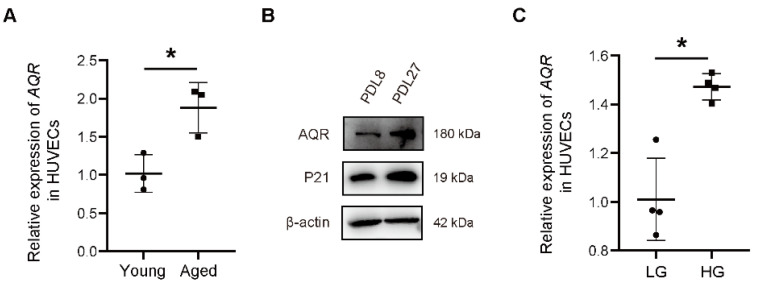

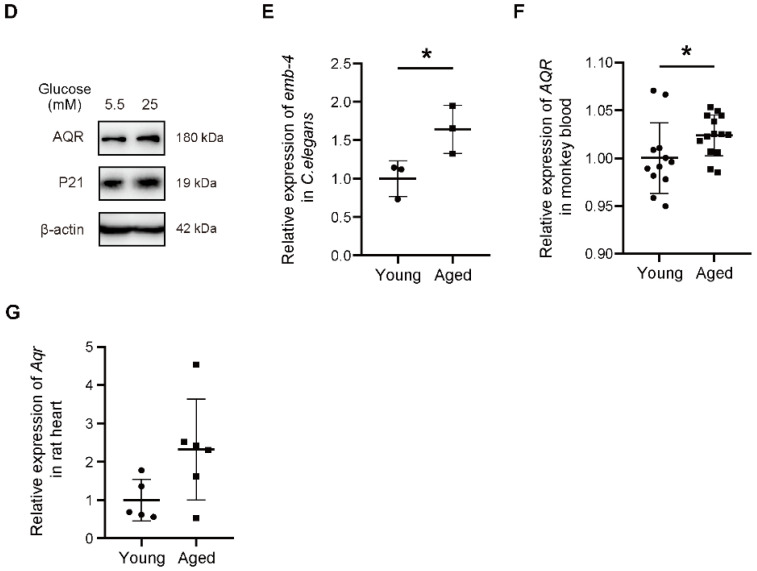
The expression of AQR is increased with aging. (**A**) qPCR analysis shows the expression of AQR was upregulated in young and aged HUVECs (Young: PDL 6, PDL 8, and PDL 9.5; Aged: PDL 26, PDL 29, and PDL 30.5; data are presented as mean ± SEM, and * referred to *p* < 0.05; relative expression levels were calculated as fold change of aged group compared with the young group). (**B**) The expression of AQR was upregulated in aged (PDL 27) generations of HUVECs at protein level, quantified by western blot, compared with the young group (PDL 8). (**C**) qPCR analysis shows the expression of AQR was upregulated in HUVECs treated with low glucose (5.5 mM) and high glucose (25 mM) for 3 days, data are presented as mean ± SEM, and * referred to *p* < 0.05; relative expression levels were calculated as fold change of high glucose group compared with the low glucose group. (**D**) The expression of AQR was upregulated in HUVECs treated with high glucose at protein level quantified by western blot, compared with the low glucose group. (**E**) Differential expression analysis of *AQR* homolog gene, *emb-4*, in *fer-15* worms during aging (Young: adult day 1; Aged: adult day 7; GEO dataset: GSE122892, Platform: GPL25147, Probe id: WBGene00001258 (Y80D3A.2 (emb-4)), *n* = 3 per group, data are presented as mean ± SEM, and * referred to *p* < 0.05; relative expression levels were calculated as fold change of old group compared to the young group). (**F**) Differential expression analysis of AQR in the whole blood of *Macaca fascicularis* (Young: 2–3 years old; Aged: 20–26 years old; GEO dataset: GSE152406, Platform: GPL16027-10477, Probe id: A_01_P1685651; *n* = 12 in the young group, *n* = 14 in the aged group; data are presented as mean ± SEM, and * referred to *p* < 0.05; relative expression levels were analyzed by the GEO2R). (**G**) Differential expression analysis of AQR in male rat hearts (Young: 2–3 months old; Aged: 20–22 months old; GEO dataset: GSE421, Platform: GPL85-10790, Probe id: rc_AA893708_at; *n* = 5 in the young group, *n* = 6 in the aged group; data are presented as mean ± SEM, and * referred to *p* < 0.05; relative expression levels were analyzed by the GEO2R).

Previously, we reported that AQR participates in glucose metabolism via the AKT/mTOR pathway, a well-known pathway involved in the process of cellular senescence. To further verify this, we detected the expression of AQR in young (PDL 6, 8, and 9.5) and aged (PDL 26, 29, and 30.5) HUVECs, and found that the expression of AQR was significantly upregulated by 85.92% ± 23.75% in aged cells (Figure 1A). Western blotting also validated its increased expression in aged (PDL 27) HUVECs compared with young HUVECs. Quality control of the samples was confirmed by the expression of a senescence-related marker P21 (Figure 1B). Furthermore, a high concentration of glucose (25 mM) elevated AQR expression by 46.25% ± 8.898% at mRNA level, compared to normal glucose condition (5.5 mM) (Figure 1C). The expression of AQR was also elevated at protein level (Figure 1D). Besides, the expression of AQR was upregulated in a publicly available RNA-seq dataset of human retinal endothelial cells treated with high glucose (25 mM) (Figure S1A). Subsequently, we retrieved a few other publicly available expression datasets (GSE122892, GSE421, and GSE152406) of aging models generated in different species and found that the expression of AQR was significantly elevated in the aged *C. elegans* and the whole blood of aged monkeys, while slightly increased in the heart of aged rats (Figure 1E–G).

### 2.2. AQR Arrests Cell Cycle at G2/M and Promotes Cellular Senescence in HUVECs

**Figure 2:**
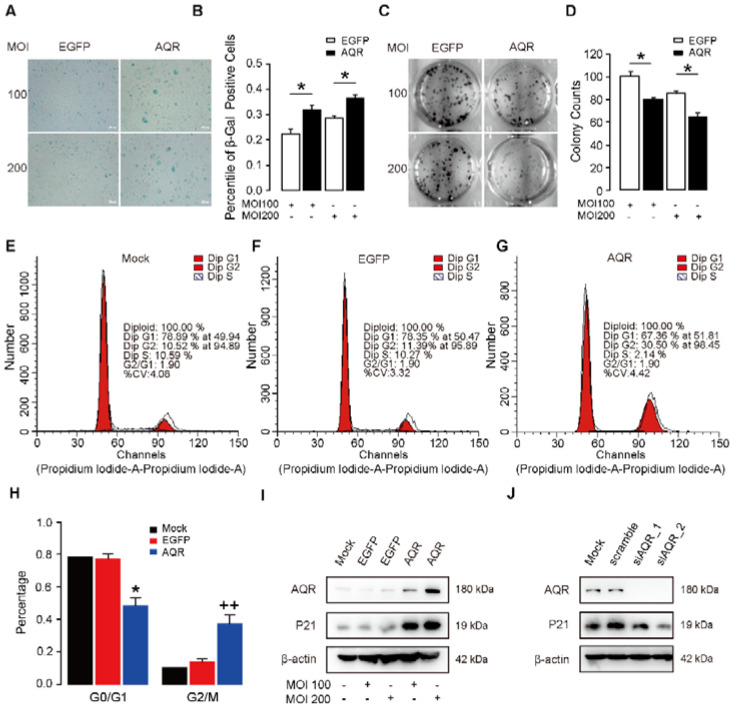
AQR arrests the cell cycle at G2/M and promotes cellular senescence in HUVECs. (**A**,**B**) Overexpression of AQR in HUVECs resulted in positive β-gal staining, (**C**,**D**) and decreased colony formation efficiency compared with the control group (EGFP). * referred to *p* < 0.05. (**E**–**G**) Overexpression of AQR increased the proportion of cells in the G2/M phase. Cell cycle analysis was performed by FACSverse. (**H**) Bar plots of cell percentages at different stages of the cell cycle, * referred to *p* < 0.05 versus the EGFP group in G0/G1; ++ referred to *p* < 0.01 versus the EGFP group in G2/M. (**I**) Western blot shows overexpression of AQR increased P21 expression in HUVECs. (**J**) Western blot shows knockdown of AQR decreased P21 expression in HUVECs.

To investigate the role of AQR in endothelial senescence, we measured the proliferation and cell cycle changes in HUVECs with a time series experiment upon the overexpression and knockdown of AQR. We overexpressed AQR using recombinant adenoviruses in HUVECs (Figure S1B) and found that it resulted in increased positive β-gal staining (Figure 2A,B) compared to the control group. Colony formation assay showed that AQR overexpression decreased the colony formation efficiency in HUVECs (Figure 2C,D). To further explore the AQR-mediated senescence in HUVECs, we performed cell cycle analyses using a flow cytometer. We found that the proportion of cell count in G0/G1 was decreased upon AQR overexpression, while that in G2/M phases was increased about 20% (Figure 2E–H). We then examined the effect of AQR on senescence biomarker, P21, in HUVECs at the protein level. AQR overexpression increased the expression of P21 (Figure 2I), while its knockdown decreased P21 expression (Figure 2J).

### 2.3. PLAU Is Co-Expressed with AQR during HUVEC Senescence

**Figure 3:**
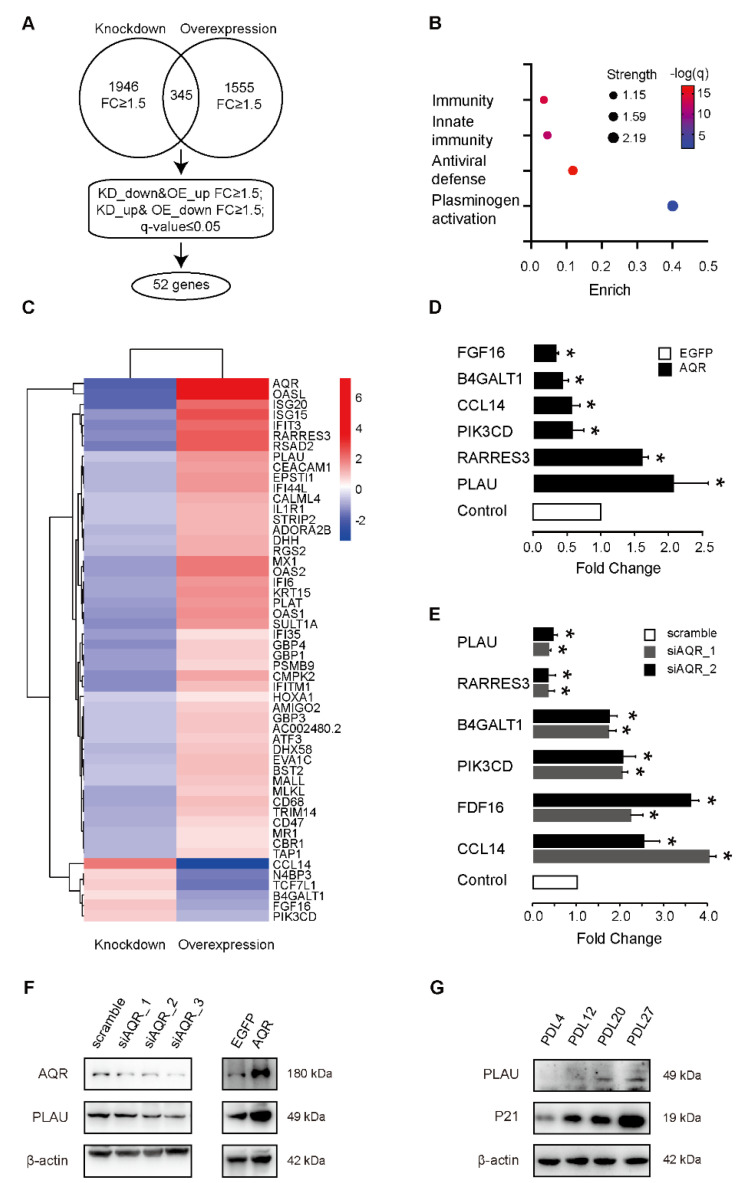
PLAU differentially co-expresses with AQR during HUVEC senescence. (**A**) Venn diagram showing the 52 differentially co-expressed genes with *AQR* corresponding to AQR overexpression and knockdown (fold change > 1.5, and q-value < 0.05). (**B**) Annotated keywords of the 52 differentially co-expressed genes. (**C**) Heatmap of the 52 differentially co-expressed genes of *AQR* according to their fold change in overexpression and knockdown of AQR. (**D**) qPCR results of the prioritized genes in the overexpression experiment. (**E**) qPCR results of the prioritized genes in the knockdown experiment. (**F**) Western blot shows AQR knockdown decreased PLAU expression, while AQR overexpression increased PLAU expression. (**G**) Western blot shows the expression of PLAU was increased in HUVECs during aging and positively co-expressed with P21. Data are presented as mean ± SEM, *n* = 3–6 per group, and * referred to *p* < 0.05 versus the scramble or EGFP control group.

To investigate the mechanism by which AQR regulated the cellular senescence, we performed RNA sequencing of HUVECs after overexpression or knockdown of AQR (Figure S1C,D). In addition to the regulation of cell proliferation, we found the enriched biological processes of differentially expressed genes with overexpressed AQR included inflammatory response, p53 signaling, and other senescence-related signaling pathways, consistent with the AQR overexpression phenotype (Figure S1E). To further validate the differential co-expression of genes with *AQR*, we combined these two datasets, including 1945 differentially expressed genes upon the knockdown of AQR and 1555 differentially expressed genes upon the overexpression of AQR (fold change > 1.5). There were 345 genes that overlapped among these two datasets, and at a cutoff value of q-value < 0.05, 52 genes were retained as differentially co-expressed with AQR, which may be potential targets and were, therefore, chosen for the subsequent annotated keywords analysis (Figure 3A,C). Enrichment analysis showed that these genes were mainly enriched in the immune response-associated pathway and plasminogen activation (Figure 3B). We then validated the 52 differentially co-expressed genes upon AQR overexpression and knockdown using qPCR. Our results showed that cell proliferation-related genes *BIRC5*, *CCL14*, *FGF16*, *GAB2*, *PIK3CD*, *PLAU*, and *RARRES3* were co-expressed with *AQR* at mRNA level (Figure 3D,E). Western blotting further confirmed that the overexpression of AQR increased the expression of PLAU, while the knockdown of AQR decreased its expression at protein level (Figure 3F). In addition, we measured the expressions of PLAU and P21 in different generations of HUVECs and found that both their expressions were upregulated in senescent HUVECs (Figure 3G).

### 2.4. PLAU Knockdown Rescues the AQR-Induced Endothelial Cell Dysfunction

**Figure 4:**
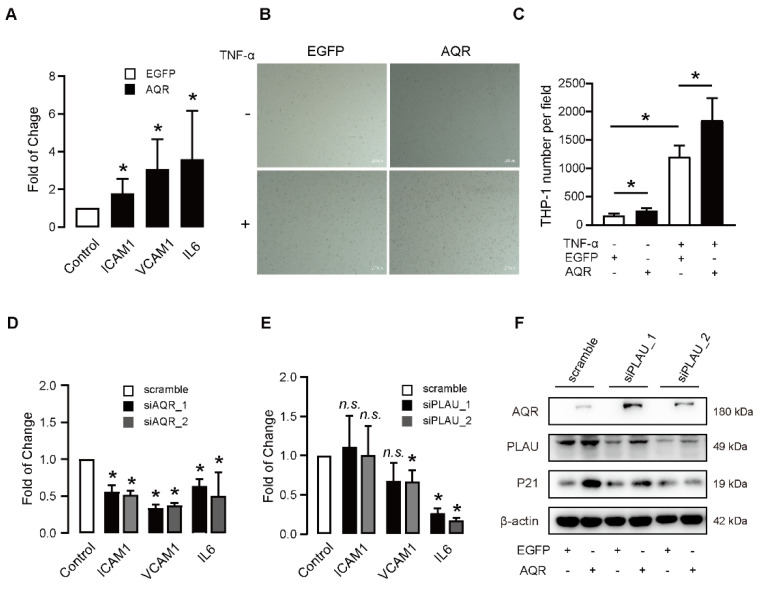
PLAU knockdown rescues the AQR-induced endothelial cell dysfunction. (**A**) qPCR results show AQR overexpression increased the expressions of ICAM1, VCAM1, and IL6 in HUVECs. Data are presented as mean ± SEM, and * referred to *p* < 0.05 versus EGFP control group. (**B**) Representative images show that AQR overexpression increased TNF-α mediated THP-1 monocytes adhesion to HUVECs. HUVECs were infected with Adv-AQR or Adv-EGFP. After 24 h, the cells were treated in the presence or absence of 10 ng/mL TNF-α for 6h and incubated with THP-1 cells for another 1 h. The attached THP-1 cells were photographed and analyzed by light microscopy. Scale bar = 200 μm. (**C**) Quantification results of TNF-α mediated THP-1 monocytes adhesion to HUVECs by overexpression of AQR. Data are presented as mean ± SEM, and * referred to *p* < 0.05 versus EGFP control group. (**D**) qPCR results show AQR knockdown decreased the expressions of ICAM1, VCAM1, and IL6 in HUVECs. Data are presented as mean ± SEM, and * referred to *p* < 0.05 versus scramble control group. (**E**) qPCR results show PLAU knockdown decreased the expressions of VCAM1 and IL6 in HUVECs. Data are presented as mean ± SEM. *n.s.* was not significant, and * referred to *p* < 0.05 versus the scramble group. (**F**) Western blot shows PLAU knockdown inhibited the upregulated expression of P21 induced by AQR overexpression.

To investigate the effects of AQR on endothelial cell function, we overexpressed and suppressed the AQR in HUVEC. IL6 is an important member of the senescence-associated secretory phenotype (SASP), while ICAM1 and VCAM1 are major adhesion molecules that indicate the function of vascular endothelial cells. We found that the expressions of VCAM1, ICAM1, and IL6 were increased upon the overexpression of AQR (Figure 4A). Given that ICAM1 plays an important role in promoting the adhesion of endothelial cells, we then determined the effects of AQR on cell adhesion function in HUVECs. We found overexpression of AQR increased the number of adherent THP-1 cells to HUVECs up to 1.5-fold, with or without TNF-α stimulation (Figure 4B,C). In addition, the knockdown of AQR decreased the expressions of VCAM1, ICAM1, and IL6 (Figure 4D), while the knockdown of PLAU only decreased the expressions of VCAM1 and IL6 (Figure 4E). To further investigate the mechanism by which AQR regulated PLAU in cellular senescence, we first overexpressed the AQR, then suppressed the expression of PLAU, and subsequently detected the expression of P21. We found knockdown of PLAU in HUVECs alleviated the upregulated expression of P21 induced by AQR (Figure 4F).

### 2.5. Functional Involvement AQR and PLAU Is Validated in Senescent Model Induced by TNF-α

**Figure 5:**
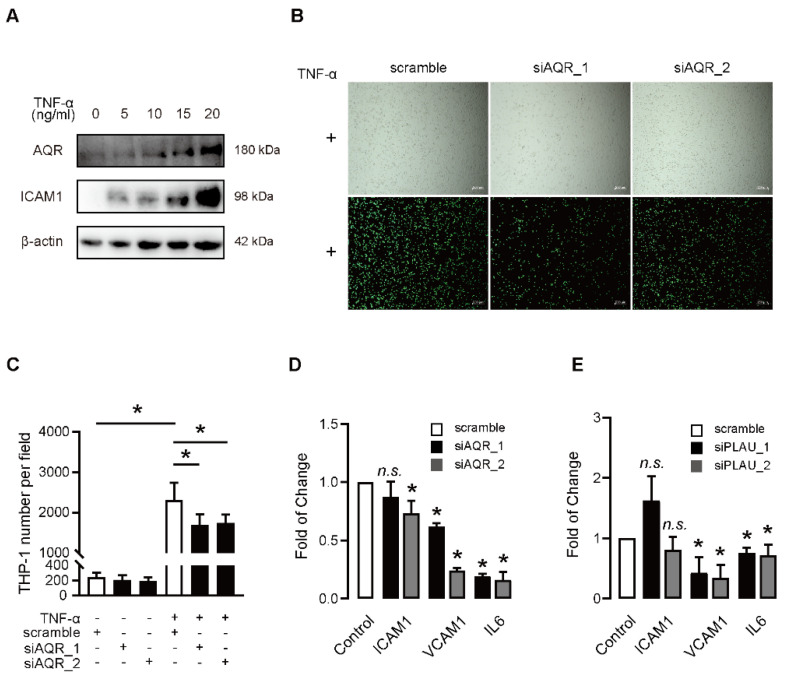
Functional involvement of AQR and PLAU is validated in the senescence model induced by TNF-α. (**A**) The expressions of AQR and ICAM1 were upregulated in response to TNF-α in a dose-dependent manner, as determined by Western blot using β-actin as an internal control. Cells were cultured for 24 h, followed by stimulation with different concentrations of TNF-α (0 ng/mL, 5 ng/mL, 10 ng/mL, 15 ng/mL, and 20 ng/mL) for 6 h. (**B**) Representative images show AQR knockdown decreased TNF-α mediated THP-1 monocytes adhesion to HUVECs. HUVECs were transfected with siAQR or scramble. After 24 h, the cells were treated in presence of 10 ng/mL TNF-α for 6 h and incubated with THP-1 cells for another 1 h. The attached THP-1 cells were photographed and analyzed by fluorescence and light microscopy. Scale bar = 200 μm. (**C**) Quantification results of TNF-α mediated THP-1 monocytes adhesion to HUVECs by silencing of AQR. (**D**) qPCR analysis shows the upregulated expressions of ICAM1, VCAM1, and IL6 in response to TNF-α were alleviated by knockdown of AQR. (**E**) qPCR analysis shows that the upregulated expressions of VCAM1 and IL6 in response to TNF-α were alleviated by knockdown of PLAU. Data are presented as mean ± SEM, *n.s.* was not significant, and * referred to *p* < 0.05 versus the scramble group.

To investigate the effects of AQR and PLAU on induced senescence, we treated HUVECs with different doses of TNF-α, a known model of induced senescence, at different time points. When treated with TNF-α, we found that expression of AQR was upregulated in a dose-dependent manner (Figure 5A). The expressions of P21 and PLAU were significantly increased by TNF-α (Figure S2A,C). qPCR results revealed that AQR and PLAU were upregulated and co-expressed in TNF-α-induced HUVECs in a time-dependent manner (Figure S2B). In response to TNF-α, the knockdown of AQR results in a reduced number of adherent THP-1 cells to HUVECs (Figure 5B,C). However, the number of adherent THP-1 cells to HUVECs was not affected by the knockdown of AQR in absence of TNF-α (Figure S2D). P65 is a hub molecule that mediates the canonical senescence pathway. The knockdown of AQR inhibited the phosphorylation of P65 (Figure S2E). Furthermore, the knockdown of PLAU decreased the expressions of VCAM1 and IL6 in TNF-α-induced HUVECs (Figure 5E), similar to that observed when AQR was knocked down (Figure 5D).

## 3. Discussion

We, in the current study, showed that the expression of *AQR*, a T2DM-susceptibility gene, was induced by aging or by high glucose. The increased AQR promoted endothelial cell senescence via PLAU-associated signaling pathways. Our study demonstrates that AQR/PLAU is a novel but critical signaling axis mediating hyperglycemia-induced cellular senescence that contributes to diabetic vascular complications. To our knowledge, this is the first study to report that a T2DM-susceptibility gene directly promotes cellular senescence.

The diabetic complications are mostly attributed to vascular dysfunction. For example, it is known that coronary artery disease, peripheral arterial disease, and stroke are macrovascular complications, while diabetic nephropathy, neuropathy, and retinopathy are microvascular complications [18]. The inflammation, ROS overproduction, and advanced glycation end-products (AGEs) caused by hyperglycemia were considered as major contributors to the complications’ etiologically, as they impair vascular function directly [19]. On the other hand, inflammation, ROS, and AGEs are well-known to promote cellular senescence, and actually, hyperglycemia-induced endothelial cell senescence was observed in vascular cells, such as endothelial cells [10,20,21,22,23], smooth muscle cells [11], endothelial progenitor cells [12], and mesenchymal stem cells [13]. These observations suggest that senescence may be serving as a shared downstream link to diabetic complications. Since the endothelial cell is critical to vascular functions, including the maintenance of vessel integrity, secretion, and participation of immune response [24], and is directly exposed to high glucose in T2DM, the senescent endothelial cell may have a profound impact on the pathogenesis of diabetic complications [20,21,22,23]. HUVEC is a widely used cellular model to investigate the regulation of endothelial function, for example, senescence and angiogenesis [25,26]. That was why we, in this study, used HUVEC to investigate the effect of AQR on cellular senescence, and we showed that high glucose induces the overexpression of the *AQR* gene which, in turn, promotes endothelial cell senescence, providing the first evidence that AQR is a novel intervenor for hyperglycemia-induced senescence.

*AQR* was initially reported as a gene responding to retinoic acid, and it contains a motif similar to RNA-dependent RNA polymerase [27]. Later, it was found that AQR was involved in RNA processing and DNA damage repair [14,28]. We also demonstrated *AQR* as a new susceptibility gene for T2DM [16]. More recently, AQR has been linked to stress scores [29]. All these reports pointed out that AQR influences multiple biological processes that are not well-characterized. Although we have demonstrated that AQR impacts glucose uptake and insulin resistance associated with the aging process, whether AQR influences cellular senescence has not yet been reported. In the current study, we presented that AQR, upregulated in different tissues across species by aging and high glucose concentrations, promotes endothelial cell senescence, providing a plausible explanation on why aging increases the susceptibility to diabetes. Cellular senescence, as a state of permanent cell cycle arrest, can occur in both G1 and G2 phase of the cell cycle, terminating the G0/G1 and G2/M progressions, respectively [30]. Two types of cell cycle arrests have distinct but partially overlapped signaling molecules [31,32]. AQR arrests the cell cycle at G2/M phase, however, the detailed mechanism needs to be further studied.

We defined PLAU as a downstream molecule of AQR-associated pathway, as knockdown of PLAU rescued AQR-induced cellular senescence-related phenotype. *PLAU* encodes a urokinase-type plasminogen activator (uPA) that binds to its receptor, uPAR, to participate in the maintenance of vascular integrity [33], tumorigenesis [34], and neuro diseases [35]. In addition, it has been well-documented that PLAU is increased during senescence and used as a biomarker for senescence, aging, and frailty, and it is also involved in the inflammatory responses [36,37,38]. By far, whether PLAU causes cellular senescence has not been reported in the existing studies. YAP upregulates the expression of PLAU [39], and YAP promotes senescence in both endothelial cells and smooth muscle cells [40], suggesting that PLAU may serve as a downstream molecule, contributing to YAP-induced vascular cell senescence. However, in another study on glioma cells, glioblastoma cells, and astrocytes, it was shown that YAP prevents senescence induced by several factors [41,42], and in cancer cells, PLAU accelerates proliferation [39,43,44,45]. These indicate that the involvement of PLAU in cellular senescence is conditional and likely cell-type dependent.

Additionally, we showed that the activation of AQR/PLAU augmented inflammation. Suppressing AQR alleviated the TNF-α-mediated upregulated expressions of IL6. The canonical NF-kB p65 pathway was also inhibited by the knockdown of AQR. PLAU has been known to be involved in the inflammatory response [46], and it has been reported that abrogating the binding of the uPA to uPAR in vivo suppressed the fibrin-associated inflammation [47]. Supportively, the increased expression of uPAR is associated with complications in diabetes patients and predicts outcomes [36,48]. Our findings indicate that the AQR/PLAU pathway regulates both inflammation and cellular senescence, likely via NF-kB-associated downstream pathway.

In conclusion, this study provides the first evidence that the T2DM susceptibility gene, AQR, promotes cellular senescence in HUVECs through PLAU-associated pathways. These findings have potential clinical implications for interventions against cellular and vascular aging.

## 4. Material and Methods

### 4.1. Cell Lines and Culture

Primary human umbilical vein endothelial cells (HUVECs) were isolated from the umbilical cord obtained from donors under an approved protocol of the People’s Hospital of Jiangxi Province (Nanchang, Jiangxi, China). Primary cells were cultured in ECM (ScienCell Research Laboratories, Carlsbad, CA, USA) supplemented with 5% fetal bovine serum (FBS), 100 mg/mL streptomycin/100 U/mL penicillin, and 1% endothelial cell growth supplement (ECGS). HUVECs at population doubling level (PDL) 12 to 18 were used for viral infection or transfection assays. THP-1 cells were cultured in RPMI medium 1640 (Gibco, Grand Island, NY, USA) containing 10% FBS.

### 4.2. siRNAs Transfection and Adenovirus Infection

HUVECs were seeded in six-well plates and transfected at 60–70% confluence with 50 nM scrambled RNA or siRNAs against human *AQR* and *PLAU* in Opti-MEM (Gibco, Grand Island, NY, USA) using Lipofectamine^TM^ 2000 (Invitrogen, Carlsbad, CA, USA). The medium was changed to ECM after transfection for 6 h, and the cells were maintained for 36 h before further experiments.

HUVECs were plated in six-well plates and infected with pAdeno-MCMV-AQR-3FLAG-IRES2-EGFP (OBIO, Shanghai, China) recombinant adenovirus in a 2-mL culture medium to overexpress AQR following the manufacturer’s guidelines. Recombinant adenovirus of pAdeno-MCMV-EGFP-3FLAG (OBIO, Shanghai, China) was used as a negative control.

### 4.3. Confocal Laser Scanning Microscopy Analysis

pAdeno-MCMV-AQR-3FLAG-IRES2-EGFP has a fluorescein-labeled EGFP by the element IRES2 to verify the validity of exogenous AQR protein expressed in the HUVECs. On the day before infection, HUVECs were seeded in 20 mm glass-bottom plates (Nest Biotechnology, Wuxi, Jiangsu, China) for confocal visualization. HUVECs were then infected with pAdeno-MCMV-AQR-3FLAG-IRES2-EGFP and pAdeno-MCMV-EGFP-3FLAG at MOI100 for 24 h. The cells were then incubated with Hoechst 33342 dye for 2 h and then observed using a two-channel confocal laser scanning microscope.

### 4.4. Quantitative RT-PCR

Total RNA was extracted from HUVECs using TRIzol Reagent (Invitrogen, Carlsbad, CA, USA) and reverse-transcribed into cDNAs using random primers (Sangon, Shanghai, China) and M-MLV reverse transcriptase (Promega, Madison, WI, USA), as per the manufacturer’s guidelines. Quantitative PCR (qPCR) was then performed using SYBR Green Real-Time PCR Master Mixes (TaKaRa, Shiga, Japan) based on the manufacturer’s protocol. The primer sequences used are listed in Table S1.

### 4.5. Western Blot Analysis

For western blotting, cells were lysed in RIPA buffer (Bmassay, Beijing, China) containing protease inhibitors (Bmassay, Beijing, China). Whole-cell lysates were separated by SDS-PAGE and then transferred to PVDF membranes (Millipore, Boston, MA, USA) pre-activated in methyl alcohol. Membranes were incubated overnight at 4 °C with primary antibodies, followed by incubation with HRP-conjugated anti-rabbit IgG or anti-mouse IgG (Cell Signaling Technology, Boston, MA, USA) secondary antibodies for subsequent detection by ECL. Primary antibodies included β-actin (Proteintech, Wuhan, Hubei, China), AQR, PLAU, ICAM1 (Abcam, Cambridge, UK), anti-NF-κB p65, anti-NF-κB p-p65, and P21 (Cell Signaling Technology, Boston, MA, USA)

### 4.6. Cell Cycle Analysis

HUVECs were cultured in 60 mm dishes. HUVECs that underwent overexpression of AQR were digested using trypsin without EDTA, washed twice with PBS, and fixed using 75% ethanol overnight at 4 °C. The ethanol was then removed using PBS and the RNase A (1% *w*/*v*) was added. HUVECs were stained using the propidium iodide (PI, 50 mg/mL) at room temperature for 15 min. DNAs labeled with PI were analyzed using the FACSverse (BD Biosciences, San Jose, CA, USA).

### 4.7. Monocyte-HUVEC Adhesion Assays

To mimic monocyte-endothelial cell interactions in vivo, THP-1 monocytes were co-cultured with HUVECs to assess their attachment in vitro. HUVECs used in the experiment were pretreated with TNF-α at a final concentration of 10 ng/mL for 6 h. After the TNF-a treatment, THP-1 monocytes were labeled with 10 μM BCECF-AM (Abcam, Cambridge, UK) at 37 °C for 30 min and added to the treated HUVEC monolayer that underwent knockdown of AQR, followed by incubation for 1 h. The cells were then gently washed with PBS to remove any unattached THP-1 cells. A fluorescence microscope was used to quantify the BCECF-AM labeled THP-1 cells adhered to the HUVECs.

### 4.8. Colony Formation Assay

HUVECs were seeded at 200 cells per well in 12-well plates. The medium was changed every 3 days, and cells were fixed and stained with 0.5% crystal violet in 75% ethanol on day 7. Colonies containing more than 50 cells were counted in each well.

### 4.9. RNA Sequencing Analysis

Cultured HUVECs were harvested 36 h after overexpression of AQR or knockdown of AQR, and total RNA was extracted using the TRIzol method. RNA libraries of knockdown of AQR were prepared to perform the single-end RNA sequencing in the Beijing Genomics Institute (BGI, Beijing, China). RNA libraries of overexpressed AQR were prepared to perform the pair end RNA sequencing on the HiSeq2500 platform (Majorbio, Shanghai, China). Fastq data were aligned to the human genome using the HISAT2 software. Differential expression analyses were performed using the Cufflinks software. Fold change > 1.5 and *p* value < 0.05 were set as the thresholds for differential expression. Enrichment analyses including gene ontologies and pathways were performed on the String website.

### 4.10. Statistical Analyses

Statistical analyses were performed in GraphPad Prism (Version 8.0.1). Quantitative data are presented as mean ± standard error of the mean (SEM). Differences between the two groups were analyzed using student’s *t*-tests. Differences among multiple groups were analyzed using ANOVA, followed by post-hoc tests. *p* < 0.05 was considered as statistically significant.

## Data Availability

Publicly available GEO datasets were analyzed in this study. GSE122892 from ‘https://www.ncbi.nlm.nih.gov/geo/query/acc.cgi?acc=GSE122892’, GSE152405 from ‘https://www.ncbi.nlm.nih.gov/geo/query/acc.cgi?acc=GSE152406’, GSE421 from ‘https://www.ncbi.nlm.nih.gov/geo/query/acc.cgi?acc=GSE421’, GSE117238 from ‘https://www.ncbi.nlm.nih.gov/geo/query/acc.cgi?acc=GSE117238’ (accessed on 13 January 2022).

## Supplementary Materials

The following are available online at https://www.mdpi.com/article/10.3390/ijms23052879/s1.

## References

[1] Forbes J.M., Cooper M.E. (2013). Mechanisms of Diabetic Complications. Physiol. Rev..

[2] Natarajan R. (2021). Epigenetic Mechanisms in Diabetic Vascular Complications and Metabolic Memory: The 2020 Edwin Bierman Award Lecture. Diabetes.

[3] Russell N.D.F., Cooper M.E. (2015). 50 years forward: Mechanisms of hyperglycaemia-driven diabetic complications. Diabetologia.

[4] Papatheodorou K., Banach M., Bekiari E., Rizzo M., Edmonds M. (2018). Complications of Diabetes 2017. J. Diabetes Res..

[5] Zhang Z.-Y., Miao L.-F., Qian L.-L., Wang N., Qi M.-M., Zhang Y.-M., Dang S.-P., Wu Y., Wang R.-X. (2019). Molecular Mechanisms of Glucose Fluctuations on Diabetic Complications. Front. Endocrinol..

[6] Qian L.-L., Liu X.-Y., Chai Q., Wang R.-X. (2018). Hydrogen Sulfide in Diabetic Complications: Focus on Molecular Mechanisms. Endocr. Metab. Immune Disord. Drug Targets.

[7] Reddy M.A., Zhang E., Natarajan R. (2015). Epigenetic mechanisms in diabetic complications and metabolic memory. Diabetologia.

[8] Cooper M.E., El-Osta A. (2010). Epigenetics: Mechanisms and implications for diabetic complications. Circ. Res..

[9] Williams M.D., Nadler J.L. (2007). Inflammatory mechanisms of diabetic complications. Curr. Diabetes Rep..

[10] Senthil K.K.J., Gokila V.M., Wang S.Y. (2017). Activation of Nrf2-mediated anti-oxidant genes by antrodin C prevents hyperglycemia-induced senescence and apoptosis in human endothelial cells. Oncotarget.

[11] Li S., Zhan J.-K., Wang Y.-J., Lin X., Zhong J.-Y., Wang Y., Tan P., He J.-Y., Cui X.-J., Chen Y.-Y. (2019). Exosomes from hyperglycemia-stimulated vascular endothelial cells contain versican that regulate calcification/senescence in vascular smooth muscle cells. Cell Biosci..

[12] Kuki S., Imanishi T., Kobayashi K., Matsuo Y., Obana M., Akasaka T. (2006). Hyperglycemia Accelerated Endothelial Progenitor Cell Senescence via the Activation of p38 Mitogen-Activated Protein Kinase. Circ. J..

[13] Yin M., Zhang Y., Yu H., Li X. (2021). Role of Hyperglycemia in the Senescence of Mesenchymal Stem Cells. Front. Cell Dev. Biol..

[14] Van Nostrand E.L., Pratt G.A., Yee B.A., Wheeler E.C., Blue S.M., Mueller J., Park S.S., Garcia K.E., Gelboin-Burkhart C., Nguyen T.B. (2020). Principles of RNA processing from analysis of enhanced CLIP maps for 150 RNA binding proteins. Genome Biol..

[15] Xie M., Roy R. (2012). Increased levels of hydrogen peroxide induce a HIF-1-dependent modification of lipid metabolism in AMPK compromised *C. elegans* dauer larvae. Cell Metab..

[16] Song C., Yan H., Wang H., Zhang Y., Cao H., Wan Y., Kong L., Chen S., Xu H., Pan B. (2018). AQR is a novel type 2 diabetes-associated gene that regulates signaling pathways critical for glucose metabolism. J. Genet. Genom..

[17] Qian H., Kowalski M.H., Kramer H.J., Tao R., Lash J.P., Stilp A.M., Cai J., Li Y., Franceschini N. (2020). Genome-Wide Association of Kidney Traits in Hispanics/Latinos Using Dense Imputed Whole-Genome Sequencing Data: The Hispanic Community Health Study/Study of Latinos. Circ. Genom. Precis. Med..

[18] Fowler M.J. (2008). Microvascular and Macrovascular Complications of Diabetes. Clin. Diabetes.

[19] Chawla R., Chawla A., Jaggi S. (2016). Microvasular and macrovascular complications in diabetes mellitus: Distinct or continuum?. Indian J. Endocrinol. Metab..

[20] Xiong Y., Wang H.-X., Yan H., Zhu S.-L., Guo S.-W., Peng W.-J., Luo D. (2021). Rutaecarpine prevents high glucose-induced endothelial cell senescence through TRPV1/ SIRT1 pathway. J. Cardiovasc. Pharmacol..

[21] Wang G., Han B., Zhang R., Liu Q., Wang X., Huang X., Liu D., Qiao W., Yang M., Luo X. (2021). C1q/TNF-Related Protein 9 Attenuates Atherosclerosis by Inhibiting Hyperglycemia-Induced Endothelial Cell Senescence Through the AMPKalpha/KLF4 Signaling Pathway. Front Pharmacol..

[22] Liu Q., Li H., Wang J., Zhong L., Chen X., Zhang R., Wang H. (2020). Glucose restriction delays senescence and promotes proliferation of HUVECs via the AMPK/SIRT1-FOXA3-Beclin1 pathway. Exp. Gerontol..

[23] Khemais-Benkhiat S., Belcastro E., Idris-Khodja N., Park S.-H., Amoura L., Abbas M., Auger C., Kessler L., Mayoux E., Toti F. (2020). Angiotensin II-induced redox-sensitive SGLT1 and 2 expression promotes high glucose-induced endothelial cell senescence. J. Cell. Mol. Med..

[24] Shao Y., Saredy J., Yang W.Y., Sun Y., Lu Y., Saaoud F., Drummer C., Johnson C., Xu K., Jiang X. (2020). Vascular Endothelial Cells and Innate Immunity. Arter. Thromb. Vasc. Biol..

[25] Lyu G., Guan Y., Zhang C., Zong L., Sun L., Huang X., Huang L., Zhang L., Tian X.-L., Zhou Z. (2018). TGF-β signaling alters H4K20me3 status via miR-29 and contributes to cellular senescence and cardiac aging. Nat. Commun..

[26] Min X., Cai M., Shao T., Xu Z., Liao Z., Liu D., Zhou M., Wu W., Zhou Y., Mo M. (2022). A circular intronic RNA ciPVT1 delays endothelial cell senescence by regulating the miR-24-3p/CDK4/pRb axis. Aging Cell.

[27] Sam M., Wurst W., Klüppel M., Jin O., Heng H., Bernstein A. (1998). Aquarius, a novel gene isolated by gene trapping with an RNA-dependent RNA polymerase motif. Dev. Dyn..

[28] Sollier J., Stork C.T., Garcia-Rubio M., Paulsen R.D., Aguilera A., Cimprich K.A. (2014). Transcription-Coupled Nucleotide Excision Repair Factors Promote R-Loop-Induced Genome Instability. Mol. Cell.

[29] Yang J. (2021). Genome-wide association studies of stress score in a Korean Cohort. Stress.

[30] White T., Deshpande N., Kumar V., Gauthier A.G., Jurkunas U.V. (2021). Cell cycle re-entry and arrest in G2/M phase induces senescence and fibrosis in Fuchs Endothelial Corneal Dystrophy. Free Radic. Biol. Med..

[31] Valentijn F.A., Falke L.L., Nguyen T.Q., Goldschmeding R. (2018). Cellular senescence in the aging and diseased kidney. J. Cell Commun. Signal..

[32] Gire V., Dulić V. (2015). Senescence from G2 arrest, revisited. Cell Cycle.

[33] Kyyriäinen J., Tapiala J., Lipponen A., Ndode-Ekane X.E., Pitkänen A. (2020). Plau/Plaur double-deficiency did not worsen lesion severity or vascular integrity after traumatic brain injury. Neurosci. Lett..

[34] Ai C., Zhang J., Lian S., Ma J., Győrffy B., Qian Z., Han Y., Feng Q. (2020). FOXM1 functions collaboratively with PLAU to promote gastric cancer progression. J. Cancer.

[35] Kyyriäinen J., Bolkvadze T., Koivisto H., Lipponen A., Pérez L.O., Ndode-Ekane X.E., Tanila H., Pitkänen A. (2019). Deficiency of urokinase-type plasminogen activator and its receptor affects social behavior and increases seizure susceptibility. Epilepsy Res..

[36] Cardoso A.L., Fernandes A., Aguilar-Pimentel J.A., De Angelis M.H., Guedes J.R., Brito M.A., Ortolano S., Pani G., Athanasopoulou S., Gonos E.S. (2018). Towards frailty biomarkers: Candidates from genes and pathways regulated in aging and age-related diseases. Ageing Res. Rev..

[37] Jiang H., Liang L., Qin J., Lu Y., Li B., Wang Y., Lin C., Zhou Q., Feng S., Yip S. (2016). Functional networks of aging markers in the glomeruli of IgA nephropathy: A new therapeutic opportunity. Oncotarget.

[38] Lehallier B., Shokhirev M.N., Wyss-Coray T., Johnson A.A. (2020). Data mining of human plasma proteins generates a multitude of highly predictive aging clocks that reflect different aspects of aging. Aging Cell.

[39] Corley S.M., Mendoza-Reinoso V., Giles N., Singer E.S., Common J.E., Wilkins M.R., Beverdam A. (2018). Plau and Tgfbr3 are YAP-regulated genes that promote keratinocyte proliferation. Cell Death Dis..

[40] Pan X., Wu B., Fan X., Xu G., Ou C., Chen M. (2021). YAP accelerates vascular senescence via blocking autophagic flux and activating mTOR. J. Cell. Mol. Med..

[41] Xu X., Shen X., Feng W., Yang D., Jin L., Wang J., Wang M., Ting Z., Xue F., Zhang J. (2020). D-galactose induces senescence of glioblastoma cells through YAP-CDK6 pathway. Aging.

[42] Xu X., Shen X., Wang J., Feng W., Wang M., Miao X., Wu Q., Wu L., Wang X., Ma Y. (2021). YAP prevents premature senescence of astrocytes and cognitive decline of Alzheimer’s disease through regulating CDK6 signaling. Aging Cell.

[43] Lin M., Zhang Z., Gao M., Yu H., Sheng H., Huang J. (2019). MicroRNA-193a-3p suppresses the colorectal cancer cell proliferation and progression through downregulating the PLAU expression. Cancer Manag. Res..

[44] Lv L., Deng H., Li Y., Zhang C., Liu X., Liu Q., Zhang D., Wang L., Pu Y., Zhang H. (2014). The DNA methylation-regulated miR-193a-3p dictates the multi-chemoresistance of bladder cancer via repression of SRSF2/PLAU/HIC2 expression. Cell Death Dis..

[45] Chen G., Sun J., Xie M., Yu S., Tang Q., Chen L. (2021). PLAU Promotes Cell Proliferation and Epithelial-Mesenchymal Transition in Head and Neck Squamous Cell Carcinoma. Front. Genet..

[46] Cunningham O., Campion S., Perry V.H., Murray C., Sidenius N., Docagne F., Cunningham C. (2009). Microglia and the urokinase plasminogen activator receptor/uPA system in innate brain inflammation. Glia.

[47] Connolly B.M., Choi E.Y., Gardsvoll H., Bey A.L., Currie B.M., Chavakis T., Liu S., Molinolo A., Ploug M., Leppla S.H. (2010). Selective abrogation of the uPA-uPAR interaction in vivo reveals a novel role in suppression of fibrin-associated inflammation. Blood.

[48] Theilade S., Lyngbaek S., Hansen T., Eugen-Olsen J., Fenger M., Rossing P., Jeppesen J.L. (2015). Soluble urokinase plasminogen activator receptor levels are elevated and associated with complications in patients with type 1 diabetes. J. Intern. Med..

