# Supplementary material for: Hyperglycemia Promotes Endothelial Cell Senescence through AQR/PLAU Signaling Axis
Source: Int J Mol Sci. 2022 Mar 7;23(5):2879. doi: 10.3390/ijms23052879 (PMC8911151; doi:10.3390/ijms23052879)
Supplement: Supplementary file 1 [file ijms-23-02879-s001.zip › ijms-1576762-supplementary.pdf]

Hyperglycemia Promotes Endothelial Cell Senescence through AQR/PLAU Signaling Axis

Yiqi Wan[1], Zhirui Liu[1], Andong Wu[1], Abdul Haseeb Khan[1], Ying Zhu[1], Shuangjin Ding[1], Xueer Li[1], Ya Zhao[1], Ximo Dai[1], Jin Zhou[1], Jiankun Liu[1], Yuanyuan Li[1], Xueting Gong[1], Man Liu[1], Xiao-Li Tian[1*]

1. Aging and Vascular Diseases, Human Aging Research Institute (HARI) and School of Life Science, Nanchang University, and Jiangxi Key Laboratory of Human Aging, Nanchang, Jiangxi 330031, China

*Correspondence: tianxiaoli@ncu.edu.cn

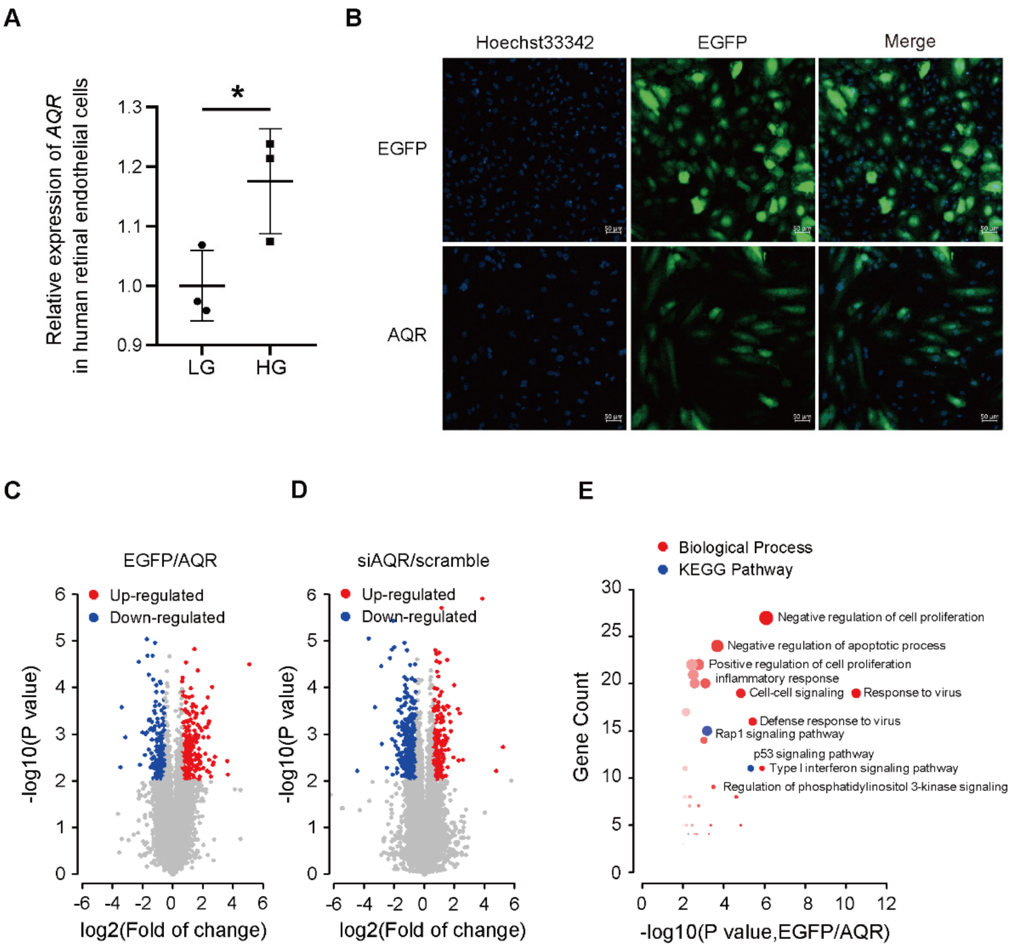

Figure S1. (A) Differential expression analysis of AQR in human retinal endothelial cells (hRECs) treated with low glucose (5 mM) and high glucose(25 mM) (LG: low glucose; HG: high glucose; GEO dataset: GSE117238, Platform: GPL21290, Probe id: ENST00000156471, n = 3 per group, data are presented as mean ± SEM, * referred to $p < 0.05$; relative expression levels were calculated as fold change of high glucose group compared with the low glucose group). **(B)** Overexpression of AQR in HUVECs increased the nuclear area. HUVECs were pretreated with Adv-EGFP or Adv-AQR for 6h, cultured for another 24 h. Nuclei were counterstained with hoechst33342 (blue). The images were obtained by fluorescence microscope and overlaid. Scale bar=50μm **(C)**

Volcano plot of the RNA sequencing data of HUVECs overexpressed with AQR (Fold change > 1.5, p-value < 0.05). **(D)** Volcano plot of the RNA sequencing data of HUVECs by silencing of AQR (Fold change > 1.5,p-value < 0.05). **(E)** Enriched biological processes and pathways of the differentially expressed genes with overexpression of AQR (Fold change > 1.5,p-value < 0.05).

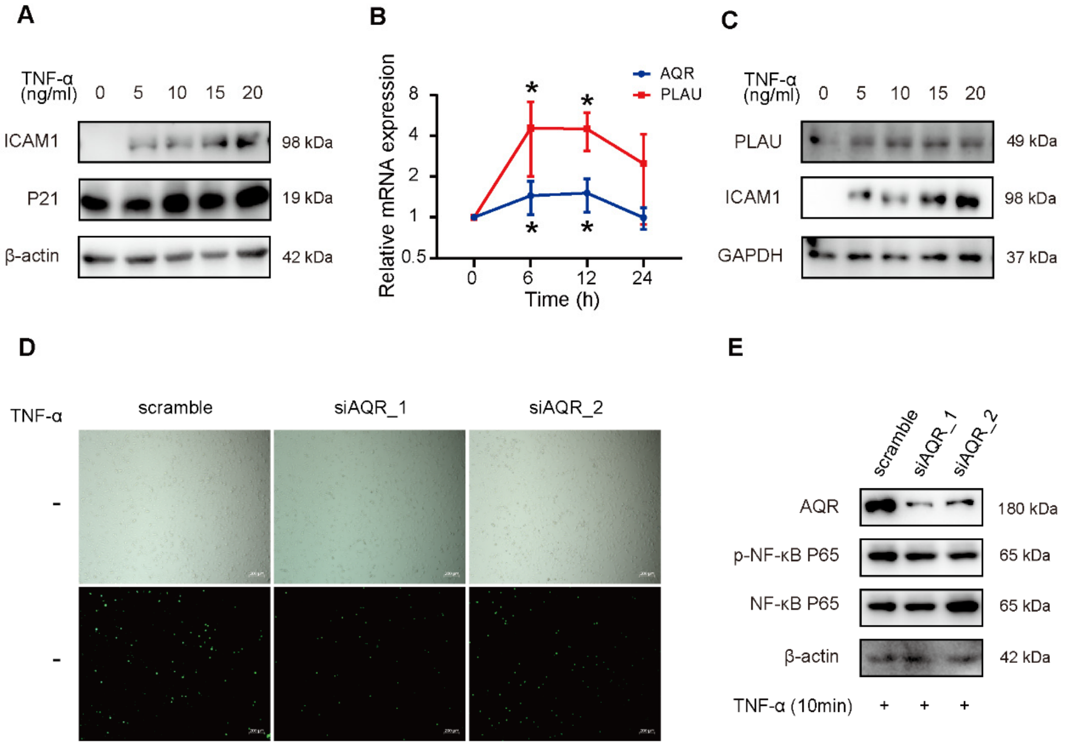

Figure S2. (A) The expression of P21 was upregulated in HUVECs treated with TNF-α, as determined by Western blot using β-actin as an internal control. Cells were cultured for 24 h, followed by stimulation with different concentrations of TNF-α (0 ng/ml, 5 ng/ml, 10 ng/ml, 15 ng/ml, 20 ng/ml) for 6 h**.** The correlation of time-course expression between AQR and PLAU in HUVECs. **(B)** qPCR analysis shows AQR and PLAU expression were both upregulated in response to 10 ng/ml TNF-α at different time points. **(C)** The expression of PLAU was also upregulated in response to TNF-α, as determined by Western blot using GAPDH as an internal control. **(D)** Cells were cultured for 24 h, followed by stimulation with different concentrations of TNF-α (0 ng/ml, 5 ng/ml, 10 ng/ml, 15 ng/ml, 20 ng/ml) for 6 h. Representative images show the effect of silencing *AQR* on THP-1 monocytes' adhesion to HUVECs. HUVECs were transfected with siAQR or scramble. 24 h later, the cells were incubated with THP-1 cells for another 1 h without treatment with TNF-α. The attached THP-1 cells were photographed and analyzed by fluorescence and light microscopy. Scale bar=200μm. **(E)** Effects of AQR silencing on the phosphorylation (Ser536) of P65 in HUVECs treated with TNF-α for a short time by western blot. Total P65 was used as an internal control.
